# Some Affections of the Esophagus

**Published:** 1914-08

**Authors:** George F. Cott

**Affiliations:** Buffalo, N. Y.


### Some Affections of the Esophagus.

By DR. GEORGE F. COTT
Of Buffalo, N. Y.

The esophagus is an elastic muscular tube about nine inches long and about one inch in calibre. It opens at the upper border of the cricoid cartilage between the fifth and sixth cervical vertebrae. The distance of the esophagus from the teeth is about six inches. Contracted portions are found at the diaphram, at the left bronchus and at its upper opening. As we have to do often with the upper esophagus we will confine ourselves to the relations of this portion of the tube. In front of the esophagus is the trachea; they are connected by areolar tissue. Anteriorly, at its lower part, where it projects towards the left it is in relation with the thyroid gland and the thoracic duct. Behind it rests on the column and the longicolli muscles, on each side is the common carotid artery and part of the lateral lobe of the thyroid gland. The recurrent laryngeal nerves lie between the esophagus and the trachea. The esophagus is composed of three coats, an external muscular consisting of longitudinal fibres, and internal muscular consisting of circular fibres, and a middle or areolar layer. Internally the esophagus is lined with mucous membrane.

Its nerve supply is derived from the esophageal plexus of the pneumogastric. The esophagus is easiest explored from the left side, therefore, when necessary an incision should be made on that side in esophagotomy.

Operations upon the esophagus is a field to which the Laryngologist has not given sufficient attention; since the advent of esophagoscopy, however, there is more of a tendency to look into diseases and operations upon this tube. To extract foreign bodies, ascertain the kind and extent of strictures, spasms, inflammation, etc., have all been under the keen eye of the laryngologist. But there is some reluctance of the part of our fraternity to treat in addition, the esophagus surgically. In order to accomplish this it is essential to bear in mind three things. Anatomy, Pathology, and Technic.

To Dr. Jackson belong the credit above all the men in this country, of developing means by which the esophagus can be handled as any other part of the body; he has done a noble work and his name will be a by-word in esophagoscopy and bronchoscopy for all time.

The esophagus is subject to pathological changes. Just as all mucous membranes they are likewise amenable to treatment. Before Jackson's admirable work we knew very little of the diseases affecting the esophagus but now diagnoses are readily made by direct inspection and treatment successfully applied where formerly this seemed physically impossible. Following is my limited experience with esophagical cases:

### DESCRIPTIVE CASES

Case 1. Man, age 30. Complained of difficult swallowing for several weeks. Office examination showed edema over arytenoids. Under ether I found it impossible to introduce the esophagoscope and desisted after a number of attempts. Next day I found out that patient had had syphilis and some painful trouble in the neck. I found his upper vertebrae were anchylosed and his head could not be straightened. He left town.

Case 2. Man, aged 60. Complained of difficulty in swallowing. Esophogoscope showed a smooth membrane healthy looking but bulging above the middle third. Gradually he swallowed with more difficulty and finally Dr. James E. King opened his stomach and in that way he was kept alive for six months. Post mortem we found a large carcinomatous mass just protruding through the esophageal mucous membrane and seemingly originating in the wall of the tube.

Case 3. Actor, age 56, looked to be 65. Difficult swallowing which gradually led him to seek relief. He was quite emaciated and coughed considerably; towards the end he could take only liquids and that was in part coughed up. Postmortem showed an opening in the tracheo-esophagus half way between the crycoid and bifurcation, large enough to admit a finger, and was probably a broken down gumma.

Case 4. Mrs. Towne, age 33. Difficult breathing and impaired swallowing. Larynx full of cancerous papillomata. About a year after operation on larynx, swallowing became impossible and gastrostomy was done by Dr. James E. King. In this way patient fed herself for two years when she succumbed to the constitutional effects of the cancer. No kind of treatment will be of benefit in the slow growing fibrous cancer; this one lasting 3 years after first operation and patient had symptoms of hoarseness five years before operation.

Case 5. Female, age 27. Felt some difficulty in swallowing and occasionally slight bleeding. A cauliflower growth was seen at the mouth of the esophagus. Microscopic examination showed epithelioma. The growth was removed by lateral esophogotomy. The esophagus was involved perhaps over an inch and another extra half inch was taken off. The wound was left open but was packed with gauze. Patient was recovering and ready to get up out of bed when on the sixth day she suddenly had a severe hemorrhage and died. No post mortem examination permitted.

Case 6. Female, age 30. Hawking up a little blood at times. Could see nothing but a large tonsil of the tongue which was removed. Several weeks later she came back with the same history, then I saw a slightly protruding growth at the mouth of the esophagus and posterior wall of the larynx. Dr. Eugene A. Smith removed the larynx and upper esophagus, patient did well for six weeks when she contracted pneumonia and suddenly died.

Case 7. Female, age 35. Swallowed false teeth which had sharp edges on aveolar frame and could not be removed without injury to the esophagus; was behind first and second ribs. Lateral esophagotomy by Dr. Kennerson and myself. Teeth removed. Fistula formed for a while through which saliva flowed, then gradually closed up. (Reported at A. M. A., New Orleans Meeting.)

Case 8. Boy, age 2½. Accidently swallowed lye. At first when I saw him the esophagus had contracted so much that only a small quantity of water would pass into the stomach. The esophagus showed a tight elongated stricture not far from the upper end through which no instrument could be passed for dilation. I then did a lateral esophogotomy but could succeed no better. Dr. James E. King then did gastrostomy for the purpose of feeding. Several times afterwards the esophoscope was introduced but with no better result. He cannot yet eat normally.

Case 9. Female, age 68, well nourished, 180 pounds, complained of slight difficulty in swallowing. There was a perceptible narrowing at upper end of the esophagus and another one several inches lower down. Here nothing serious being suspected, because of the short duration, perfectly healthy appearance, no cachexia, nor loss of weight. An electric bougie was passed and gradually both strictures admitted the instrument. Immediately afterwards she swallowed half a glass of water with considerable comfort. A second sitting was had several days later; during the operation she collapsed and had to be taken home in a carriage. She died suddenly during the night. Post mortem, was found a recent pericarditis. A large carcinomatous mass at the cardiac end of the stomach in the esophagus and another mass at the upper end of the tube. The cause of death seems to have been caused by the electrode producing pericarditis and consequently profound shock from which the patient failed to rally.

Case 10. Little girl, age 3 months, swallowed a small clasp pin, (the kind used by ladies to fasten the collar). X-Ray located it in upper esophagus, closed. I could easily feel it with the index finger, but when the short tube was introduced it had disappeared. Another picture located it in the stomach, but we did not interfere with its progress; three more pictures located it in different parts of the intestinal tract; it finally passed out without causing any damage.

Case 11. A little child, age 1½, accidently swallowed a small open safety pin with the point downward. It was located just within the upper esophageal opening but when the esophagoscope was introduced it had disappeared. In some unaccountable way it passed into the stomach because the tube met with no obstruction. The next picture showed it in the stomach engaged in the pylorous the open pin towards the exit. Upon consultation it was deemed best to remove it by gastrotomy which Dr. James E. King performed. He found it fastened in the pylorus.

Case 12. Child, age 2½, swallowed a brass key about one inch long. I found it tightly imbedded just within the esophagus, but found it very difficult to locate with the tube; after several ineffectual efforts I removed it by a lateral opening. The child died the same night, no doubt of shock, for I learned afterwards that another physician had worked for an hour trying to remove the key with forceps.

Case 13. A child, age 4, swallowed a "nickel" six days before coming to the hospital. I could locate it immediately below the upper entrance of the esophagus, but could not budge it from its fastened position. It could not be seen through the Brüning tube, nor could I engage it while observing it with the flouroscope. This I also removed through a lateral opening. Recovery.

Case 14. Child, age 5. Swallowed a nickel seven days before. X-Ray showed it imbedded in the mucous membrane. I could feel it with a probe through the tube but could not see or engage it. A coin extractor finally removed it readily.

Case 15. Man, age 68. Difficult swallowing for many years. X-Ray showed two distinct strictures in the esophagus with diverticulae. At times swallowing was difficult, due apparently to spasms at one of the strictures which passed off in a few minutes and food passed down again, or he would vomit what he had eaten and at other times in the middle of a meal he was unable to swallow further and had to rest for several hours. Once or twice a year there would be inflammation of the esophagus when he would have to take to his bed and could pass nothing for several days, then recover and be in apparent perfect health. If permission were given dilation might be of benefit.

Case 16. Carpenter, age 58. Arterio-sclerosis, somewhat emaciated, spasmodic cough often keeping him awake nights. Noticed some impairment in swallowing for six months.

Sought treatment on account of cough and incidentally mentioned the difficult swallowing. He located the source of cough in the region of the cricoid. Larynx looked healthy, but moderately large lingual tonsil which was removed, with apparent relief. A few days later cough recurred as bad as ever. Small bougies met some obstruction immediately within the esophagus. Ether was administered and the esophagoscope introduced. Mucous membrane normal, but bulged just below upper opening posteriorly. Diagnosis: Probably carcinoma in wall of esophagus. Cough reflex through pneumogastric. Could not follow up the case.

Several cases of functional spasma of the esophagus were seen in which the mucous membrane of the esophagus was intensely red, but no obstruction observed; it was characterized by pain in some part of the esophagus which passed off after some minutes duration and liquids passed down slowly or were detained for a short time then passed into the stomach. X-Ray and esophagoscope showed nothing and recovery was complete.

Three or four cases were observed where the patient complained of difficult swallowing. Here whenever permission was obtained the esophagus located a hard mass about half way down to the stomach. A tentative diagnosis of carcinoma was made and all died but one, a man aged 62, who is still alive and enjoying quite good health with, however, difficult swallowing, but fairly well nourished, eating generally soft food. He has not grown any worse during the last five years.

This patient was examined by Dr. Jackson who concurred in the opinion that it was carcinoma. X-Ray showed a distinct growth bulging into the esophagus. Five years later patient died suddenly of heart trouble.

If one could see many cases of esophageal obstruction or foreign bodies much better treatment and results could be obtained, but the limited number of cases is a great hindrance to the laryngologist and he will often fail in getting results in which men like Dr. Jackson easily succeed, for the esophagus is frequently the seat of disease just as any other part of the body as has been shown by the excellent work of Dr. Jackson, and is just as amenable to treatment.

The field, however is not a remunerative one, and requires considerable dexterity in manipulation. It may gradually be confined to specialists in every large city who will merit the support of every practitioner; then we may look for uniformly good results as to the diagnosis and treatment.

1195 Main Street.

